# Minutes of the 28th Annual Meeting of the Mississippi Valley Dental Society

**Published:** 1872-05

**Authors:** Frank A. Hunter

**Affiliations:** Rec. Sec’y.


### PROCEEDINGS OF SOCIETIES.

### Minutes of the 28th Annual Meeting of the Mississippi Valley Dental Society, Beginning Wednesday, March 6th, 1872.

WEDNESDAY, A. M.

The Society was called to order by the President, and opened by the reading of a portion of the Holy Scriptures, and Prayer by the Rev. Dr. Jeffreys, of Cincinnati, after which the roll was called, and a committee apointed to correct the roll, consisting of Drs. C. R. Taft, C. M. Wright and B. D. Wheeler.

Minutes of the last annual meeting were read and approved. The Executive Committee handed in their report, and, on motion, a regular reporter was engaged to take the proceedings of the meeting. Neither the Committee on Ethics or Appliances made any report. On motion of Dr. Taft, the time for meetings was adopted: Morning, from 9 till 12; Afternoon, 2 till 5½; Evening, 7½ till 10.

The Committee on membership proposed the following names: W. G. Cummins, Sturgis, Mich.; J. R. Clayton, J. A. Rowsey, Toledo; W. J. Cooley, of Athens, O.; Frank A. Hunter, of Cincinnati; S. E. Harryman, of Lawrenceburgh. A Committee of three, composed of Drs. Taft, Morgan and McKellops, was appointed to prepare letters of recognition for Prof. C. M. Wright, who goes to Switzerland; Dr. N. Williams, who goes to Italy; and Dr. D. Phillips, who goes to South America.

On motion, Drs. Williams and Harryman were appointed to codify the constitution and By-Laws for ready reference and report at the present meeting.

Adjourned.

AFTERNOON SESSION.

Society met at 2 o'clock. Minutes read and approved. Dr. J. G. Cameron, Treasurer, made his report for two years. Drs. Keely and Taylor were appointed to audit the report.

The Pres., Dr. H. A. Smith, delivered an address on Dental Legislation, and was requested by the Society to place it in the hands of the publishing committee.

The Subject for discussion: "What are the legitimate requirements and expectations of the dentist, in view of the necessities that exist, and the advantages available?"—was introduced and discussed by Drs. Taft, McCollum, Watt, Morgan, Rawls, Wright, McKellops and Rosson.

Dr. McCullom's paper was requested for the publishing committee.

Adjourned.

THURSDAY, A. M.

Society called to order at 9 o'clock. Minutes read and approved. Committee on codifying the constitution reported. Report accepted and committee discharged.

Report of committee on revising roll read by Dr. C. R. Taft. Dr. McKellops proposed that the same committee be instructed to prepare a circular notifying delinquents of the resolution passed in regard to the roll, which was so ordered.

The next subject in order was—"What are the obstacles in the way of a complete fulfillment of professional responsibility, and how are they to be overcome?"—discussed by Drs. Morgan, Taft, McCullum, H. R. Smith, J. Taylor, Horton and Osmond.

Adjourned.

AFTERNOON SESSION.

Afternoon was devoted to Dr. Corydon Palmer's demonstrations.

Adjourned.

FRIDAY, A. M.

Society met at 9 o'clock. Miscellaneous business in order. Committee on membership reported the following names: Drs. A. F. Emminger, J. T. Dennis, D. C. May, D. Gale French, W. F. Harbaugh, J. B. Welch, D. W. Clancy, J. H. Warner; all of whom were elected.

Mrs. Marie Grubert, of Berlin, was elected a member of the

Society; her name was also referred to the committee appointed yesterday for a letter of recognition and compliment from the Society.

It was resolved that the sum of $25 00 be given to Dr. Corydon Palmer, as a slight token of appreciation of his efforts in behalf of the S ociety.

The following bills were read and accepted: Miss. Val. Dent. Society—To J. Tatt, for issuing notices of meeting, $5 50; Miss. Val. Dent. Society—For reporting discussions, $35 00; Miss. Val. Dent. Society—For care of rooms, $7 00.

Dr. W. F. Morrell, New Albany, Ind., read an interesting paper on the second subject of discussion, viz: "What are the obstacles in the way of a complete fulfillment of professional responsibility, and how are they to be overcome?" Referred to publishing committee.

The 4th and 5th topics, viz: "What is the present status of the profession in ability to protect, rescue and save the natural teeth?" and ,' What are the means requisite for securing the largest measure of success in the preservation of the teeth?"—were discussed by Drs. Osmond, Morgan, Taft, Watt, McClelland, McCullum, Keely, Knapp, Horton and Baxter.

The election of officers for the ensuing year resulted as follows: Dr. Rehwinkel, Pres.; Dr. Marie Grubert, Vice Pres.; Dr. Frank A. Hunter, Rec. Sec.; Dr. Will Taft, Cor. Sec.; Dr. J. G. Cameron, Treas.

Dr. Corydon Palmer was elected a member of the Society.

The exhibition of Appliances was announced to take place at two o'clock, and the Society adjourned to meet at 2½ o'clock.

AFTERNOON SESSION.

Society called to order by the Pres. Minutes read and approved. Dr. McClelland, of Louisville, was requested to address the meeting in regard to Rose Pearl.

The President elect was conducted to the chair and made a few remarks. Dr. Marie Grubert expressed thanks for the honor conferred upon her by the election. Dr. Watt offered the report of the committee on appliances, which was accepted and referred to the publishing committee.

A vote of thanks was tendered Dr. Baxter for the valuable device of his magnifying lens; and to Dr. Watt for his improved drill.

The following delegates were elected to represent the Society in the American Dental Association: Drs. Rowsey, Toledo; J. F. Dennis, Washington, O.; R. S. Evans, Toledo; E. C. Sloan, Ironton, O.; A. F. Emminger, Columbus; E. Osmond, Cincinnati; J. H. Warner, Cincinnati; J. W. Baxter, Vevay, Ind.; J. A. Stipp, Sydney; D. W. Clancey, Cincinnati; and Mrs. Marie Grubert, Berlin.

The name of Dr. Isaac Knapp, of Ft. Wayne, was proposed for membership, and he was elected by acclamation.

The next subject: "What is the present status of the profession in ability to supplement the loss of the natural teeth, and the defects occasioned thereby; and what are the impediments in the way of higher attainments in this direction?"—was discussed by Drs. Palmer, Taft, Hunter, Osmond, Wright, Horton and H. A. Smith.

Dr. Palmer was requested to explain his mode of articulating artificial teeth, but, before concluding, the hour of adjournment arrived, and the balance of the lecture was postponed until the Evening Session.

### EVENING SESSION.

The meeting was called to order by Pres. Rehwinkel at 8 o'clock. The following committees were appointed:

Executive Committee.—H. A. Smith, Cincinnati; W. P. Horton, Cleveland; Isaac Knapp, Ft. Wayne.

On Publication—J. Taft, Cincinnati; H. R. Smith, Cincinnati; G. W. Keely, Oxford.

On Membership—P. G. C. Hunt, Indianapolis; D. W. Clancey, Cincinnati; W. F. Morrell, New Albany.

On Ethics—W. H. Morgan, Nashville, Tenn.; E. C. Sloan, Ironton, O.

On Appliances—Merit Wells; R. A. Mollyeaux; Corydon Palmer.

Dr. Palmer concluded his lecture on articulating artificial teeth.

The following was offered by Dr. Rehwinkel:

*Resolved*, That the delegates of this Society be instructed to urge upon the American Dental Association to memoralize the Congress of the United States, setting forth the necessity of appointing competent Dental Surgeons to the Army and Navy of the United States.

Carried.

The following Preamble and Resolutions were offered by Dr. Clancey:

WHEREAS, The knowledge and means that our special department of Surgery obliges us at the present time to arrest the ravages of diseases of the teeth, and preserve them to usefulness, and, inasmuch as the practice is still not uncommon within the pale of our profession to sacrifice the work of the Creator to that of the artizan;

*Resolved*, That this Association regard it as criminal and unprofessional to remove any of these important organs, under any circumstances whatever, that by the skill of the Dental Surgeon, there is a probability of saving it to the use and comfort of its possessor.

*Resolved*, That we have no right to yield to the patient, in his or her weakness for false teeth, and hold in contempt any engaged in the practice of Dentistry who would discourage his patient in having teeth treated if they are at all amenable to treatment, with the pretense that artificial teeth to him are inevitable, and that he might as well have them first as last.

On motion these were indefinately postponed.

On motion adjourned till next annual meeting.

FRANK A. HUNTER, Rec. Sec'y.

